# Diagnostic utility of inflammatory markers in formalin‐fixed and paraffin‐embedded muscle biopsies for idiopathic inflammatory myopathies

**DOI:** 10.1111/his.70191

**Published:** 2026-06-10

**Authors:** Chinnawut Suriyonplengsaeng, Jariya Waisayarat

**Affiliations:** ^1^ Department of Anatomy, Faculty of Science Mahidol University Bangkok Thailand; ^2^ Clinical Anatomy Research and Education Laboratory (CARE Lab), Faculty of Science Mahidol University Bangkok Thailand; ^3^ Department of Pathology, Faculty of Medicine Ramathibodi Hospital Mahidol University Bangkok Thailand

**Keywords:** diagnostic immunohistochemistry, formalin‐fixed and paraffin‐embedded, idiopathic inflammatory myopathy, muscle biopsy, snap‐frozen section

## Abstract

**Objective:**

This study aimed to develop a reproducible immunohistochemistry method for formalin‐fixed, paraffin‐embedded (FFPE) muscle sections and to assess its diagnostic utility for idiopathic inflammatory myopathies (IIM) in settings where snap‐frozen muscle biopsy is unavailable.

**Methods:**

Twenty cases were analysed: dermatomyositis (*n* = 5), inclusion body myositis (*n* = 2), immune‐mediated necrotizing myopathy (*n* = 5), overlap myositis (*n* = 1), unspecified myopathy (*n* = 3), mitochondrial myopathy (*n* = 1), LGMD R2 dysferlin‐related muscular dystrophy (*n* = 1), facioscapulohumeral muscular dystrophy (*n* = 1), and neurogenic muscle change (*n* = 1). Immunohistochemistry was performed on 3‐μm FFPE muscle sections using heat‐induced antigen retrieval with citrate/EDTA buffer and overnight incubation at 4°C with antibodies against MHC‐I, MHC‐II, MAC, MxA, and p62. Frozen sections were analysed in parallel for comparison.

**Results:**

Comparable staining intensities and patterns for MHC‐II, MxA, and p62 were achieved in FFPE muscle sections. Although discrepancies between frozen and FFPE sections occurred in eight cases, each involving a single marker, no statistically significant differences in immunohistochemical results were observed for MHC‐II (*P* = 0.4795), MxA (*P* = 0.4795), and p62 (*P* = 0.6171). Concordance between methods was high, reaching 90% for MHC‐II and MxA and 80% for p62. These isolated discrepancies may reflect variability in antigen expression associated with the segmental and patchy involvement characteristic of IIM. However, MHC‐I and MAC staining were unsuccessful in FFPE sections.

**Conclusions:**

Successful immunolabelling of MHC‐II, MxA, and p62 was achieved in FFPE muscle sections. These findings indicate that several diagnostically informative markers for IIM can be reliably assessed in FFPE muscle, providing a practical diagnostic option in resource‐constrained settings.

## Graphical abstract

Formalin‐fixed, paraffin‐embedded (FFPE) muscle tissue supports robust immunohistochemical detection of MHC II, MxA, and p62 with performance comparable to frozen sections. This approach reliably identifies the pathological signatures of inclusion body myositis, dermatomyositis, immune‐mediated necrotizing myopathy, and overlap myositis, enhancing the accessibility of idiopathic inflammatory myopathy diagnosis.

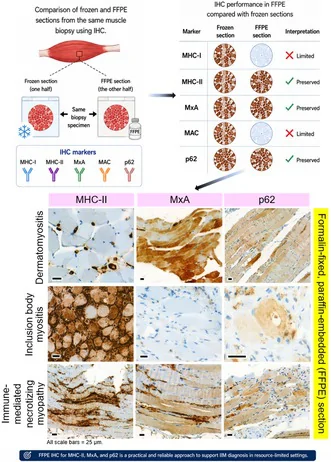

**Abbreviations**
DM: dermatomyositis
FFPE: formalin‐fixed, paraffin‐embedded
IBM: inclusion body myositis
IHC: immunohistochemistry
IIM: inflammatory myopathies
IMNM: immune‐mediated necrotizing myopathy
MAC: membrane attack complex
MHC‐I: major histocompatibility complex class I
MHC‐II: major histocompatibility complex class II
MxA: myxovirus resistance protein A
OM: overlap myositis

## Introduction

Idiopathic inflammatory myopathies (IIM) are a diverse group of immune‐mediated muscle diseases characterized by muscle inflammation and progressive muscle weakness. Recent consensus has refined IIM classification into five distinct subtypes, including dermatomyositis (DM), inclusion body myositis (IBM), immune‐mediated necrotizing myopathy (IMNM), anti‐synthetase syndrome, and overlap myositis (OM).^1^, ^2^, ^3^, ^4^, ^5^, ^6^ Polymyositis, once regarded as a distinct entity, is increasingly being reclassified under other myopathy subtypes and is now considered an obsolete diagnosis.^1^, ^2^, ^3^, ^4^ Clinical information, autoantibody profile, and muscle biopsy are essential in the diagnosis of IIM. Snap‐frozen muscle biopsy is considered the gold standard, as it preserves enzyme activity and tissue antigenicity critical for accurate histopathological assessment.^7^ However, its limited availability in resource‐constrained settings remains a significant challenge. In Thailand, snap‐frozen muscle biopsy is currently offered at only a few tertiary care hospitals in Bangkok,^8^, ^9^ despite the country comprising 77 provinces. This limited accessibility restricts patients' access to timely and accurate diagnosis, ultimately delaying appropriate treatment. In contrast, immunohistochemistry (IHC) using formalin‐fixed, paraffin‐embedded (FFPE) muscle tissue is far more accessible. However, studies investigating the diagnostic potential of FFPE muscle specimens in IIM remain limited. A similar challenge exists in muscular dystrophy diagnosis, which prompted us to develop a reliable and reproducible IHC protocol using FFPE muscle tissue to detect sarcolemmal membrane‐associated proteins for diagnosing common muscular dystrophies.^10^ Building on this success, we aim to overcome similar limitations in IIM diagnosis by developing a sensitive and reproducible IHC method for detecting inflammatory markers in FFPE muscle samples that is comparable to the snap‐frozen technique. This study focused on major histocompatibility complex class I (MHC‐I), major histocompatibility complex class II (MHC‐II), membrane attack complex (MAC or C5b‐9), myxovirus resistance protein A (MxA), and p62, which reveal aberrant MHC expression, complement deposition, type I interferon activity, and autophagic vacuoles, respectively.^11^ The second objective was to evaluate the diagnostic utility of these markers for IIM using FFPE muscle biopsy specimens.

## Materials and Methods

### Study Design and Case Selection

Ethical approval for this study was granted by the Human Research Ethics Committee, Faculty of Medicine, Ramathibodi Hospital, Mahidol University (COA. MURA2022/301), including permission to use biobank tissue in lieu of informed consent. The study included 20 patients diagnosed as follows: DM (*n* = 5), IBM (*n* = 2), IMNM (*n* = 5), OM (*n* = 1), unspecified myopathy (*n* = 3), mitochondrial myopathy (*n* = 1), LGMD R2 dysferlin‐related muscular dystrophy (formerly LGMD2B) (*n* = 1), facioscapulohumeral dystrophy (*n* = 1), and neurogenic change of muscle (*n* = 1). Diagnoses were previously determined based on clinical presentation, histopathological evaluation, and immunophenotyping of snap‐frozen muscle tissue. Detailed case information was presented in Table 1.

**Table 1:** Clinical profiles of 20 patients, including sex, age, and diagnosis, with a comparative immunohistochemical analysis between frozen and FFPE sections

Case	Sex	Age (years)	Diagnosis	Frozen section					Formalin‐fixed, paraffin‐embedded section				
				MHC‐I	MHC‐II	MAC	MxA	p62	MHC‐I	MHC‐II	MAC	MxA	p62
1	F	61	Dermatomyositis	+	−	+	++, PFA	+++	Unsuccessful Staining	−	Unsuccessful Staining	++, PFA	+++
2	M	55	Dermatomyositis (Tif 1 gamma)	−	−	−	−	−		−		−	+
3	F	60	Dermatomyositis	+	−	+	−	++		−		+++, PFA	++
4	F	7	Dermatomyositis, juvenile	+	++, PFA	+	−	++		++, PFA		−	++
5	F	14	Dermatomyositis, juvenile	+	++	+	+++, PFA	+++		−		+++, PFA	+++
6	M	76	Inclusion body myositis	+	+++	−	−	+++		+++		−	+++
7	M	67	Inclusion body myositis	−	+++	−	−	++		+++		−	++
8	M	65	Immune‐mediated necrotizing myopathy	−	−	−	−	++		+++		−	+++
9	F	10	Immune‐mediated necrotizing myopathy	+	−	+	−	++		−		−	−
10	F	14	Immune‐mediated necrotizing myopathy	+	−	+	−	+++		−		−	+++
11	F	79	Immune‐mediated necrotizing myopathy	+	+++	+	++	+++		++		++	+++
12	M	78	Immune‐mediated necrotizing myopathy	+	−	+	++	+++		−		−	+++
13	F	56	Overlap myositis	+	−	+	−	++		−		−	++
14	M	61	Unspecified myopathy	−	−	−	−	++		−		−	+++
15	F	61	Unspecified myopathy	−	−	−	−	−		−		−	+++
16	F	16	Unspecified myopathy	−	−	−	−	+		−		−	+
17	M	31	Mitochondrial myopathy	−	−	−	−	+		−		−	+
18	F	42	LGMD R2 dysferlin‐related muscular dystrophy (formerly LGMD2B)	−	−	−	−	++		−		−	++
19	M	68	Facioscapulohumeral dystrophy	−	−	+	−	+++		−		−	+++
20	M	67	Neurogenic change of muscle	−	−	−	−	+++		−		−	−

Result cells with a coloured background indicate discordant findings.

F: Female; M: Male; PFA: Perifascicular atrophy.

### Tissue Processing

Muscle biopsies were performed under local anaesthesia, targeting either the quadriceps femoris or the biceps brachii, depending on clinical indication. Fresh tissue samples were promptly transported to the pathology laboratory to preserve integrity. Each specimen was bisected: one portion underwent snap freezing, while the other was fixed in 10% neutral‐buffered formalin and processed into paraffin‐embedded blocks.

### Immunohistochemistry

FFPE muscle sections were cut at 3 μm from each paraffin block, mounted on glass slides, and incubated at 60°C for at least 1 h. Slides were deparaffinized, rehydrated, and subjected to heat‐induced antigen retrieval at 97°C for 20 min in citrate buffer followed by 97°C for 20 min in EDTA buffer. After rinsing in distilled water, endogenous peroxidase activity was blocked with 5% hydrogen peroxide for 30 min. Non‐specific background staining was minimized using Bond Primary Antibody Diluent (Ventana) for 15 min at room temperature. Slides were washed with EnVision FLEX Wash Buffer (1×) (Dako). Primary antibodies against MHC‐I, MHC‐II, MAC, MxA, and p62 (manufacturer, clone, catalogue number, and dilution detailed in Table 2) were incubated overnight at 4°C. Immunostaining was subsequently processed using automated staining platforms (Ventana for MHC‐II, MxA, and p62; Autostainer Link 48, Dako, for MHC‐I and MAC) according to the manufacturers' protocols. Slides were then counterstained, dehydrated, cleared, and coverslipped.

**Table 2:** Details of the antibodies and staining patterns

Antibody	Manufacturer	Clone [catalogue number]	Dilution for frozen section	Dilution for FFPE section	Staining pattern
MHC‐I	NeoMarker	W6/32 [MS‐1218‐P]	1:15,000 (Ventana)	1:50 (Dako)	Sarcolemmal and sarcoplasmic staining
MHC‐II	Dako	CR3/43 [M0775]	1:100 (Ventana)	1:50 (Ventana)	Sarcolemmal and sarcoplasmic staining; Perifascicular or non‐perifascicular area
MAC (c5b‐9)	Abcam	aE11 [ab66768]	1:300 (Ventana)	1:50 (Dako)	Sarcolemmal staining or endomysial capillary thrombotic pattern
MxA	EMD Millipore	M143 (CL143) [MABF938]	1:300 (Ventana)	1:200 (Ventana)	Sarcoplasmic staining; Perifascicular or non‐perifascicular area
p62 (SQSTM1)	Santa cruz	D‐3 [sc‐28359]	1:200 (Ventana)	1:200 (Ventana)	Sarcoplasmic staining; Fine granular or clumping staining in rimmed vacuoles

Frozen sections were prepared as controls. Fresh muscle specimens were rapidly frozen in isopentane precooled in liquid nitrogen (−150°C) and subsequently stored at −80°C. Cryostat sections (10 μm) were cut, mounted on glass slides, and air‐dried at room temperature. IHC was performed without prior fixation or antigen retrieval. Primary antibodies diluted in Bond Primary Antibody Diluent (Leica) were applied for 40 min. Subsequent immunostaining was performed on an automated Ventana platform according to the manufacturer's protocols. Slides were then counterstained with haematoxylin, dehydrated, cleared, and coverslipped.

All five clones were selected because they are routinely employed in our diagnostic practice. MHC‐I and MAC have been manufacturer‐validated exclusively for frozen tissue sections, whereas MHC‐II, MxA, and p62 are validated for use in both frozen and FFPE specimens.

Positive controls for FFPE and frozen sections were included alongside each tissue section on the glass slide. Each case number was blinded and randomly reordered for IHC interpretation. IHC results were initially evaluated independently, without clinical information. Positive immunoreactivity criteria were defined as shown in Table 2. The intensity of immunoreactivity was graded as follows: – (negative), + (weak), ++ (moderate), and +++ (strong). Subsequently, the IHC findings from either FFPE or frozen muscle sections were correlated with the clinical context to render a diagnosis. This diagnostic approach was applied separately to FFPE and frozen sections. A comparison of IHC results between FFPE and frozen sections was performed.

### Statistical Analysis

Differences in immunohistochemical results between frozen and FFPE muscle sections were evaluated using McNemar's test with Edwards continuity correction for paired categorical data (*P*‐value <0.05 considered statistically significant).

## Results

A comprehensive IHC characterization of MHC‐I, MHC‐II, MAC, MxA, and p62 using frozen and FFPE sections in all 20 cases was meticulously summarized in Table 1. Multiple antibody dilutions were evaluated for FFPE sections to determine the optimal staining conditions. Successful staining of MHC‐II, MxA, and p62 in FFPE sections was achieved at the optimal antibody dilution of 1:50, 1:200, and 1:200, respectively (Table 2). In contrast, MHC‐I and MAC were not demonstrable in any FFPE sections, even at a dilution of 1:50.

**Figure 1:**
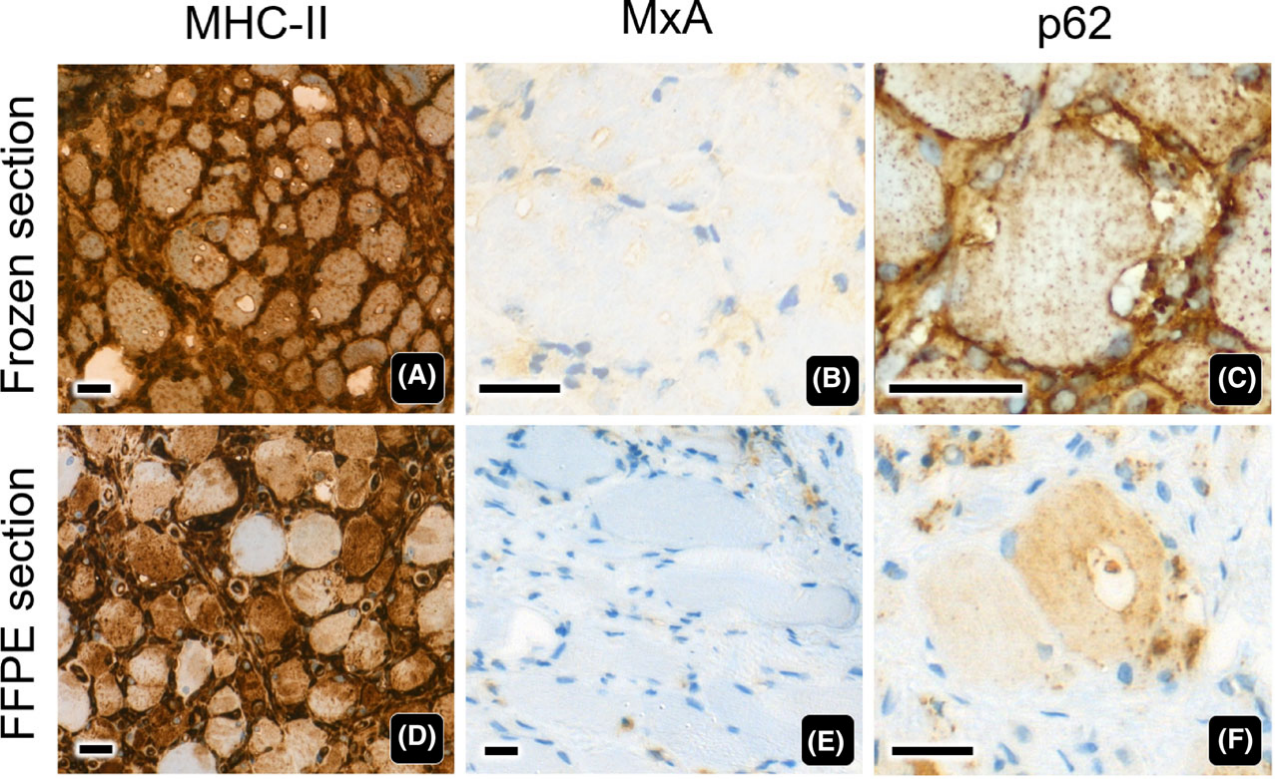
Inclusion body myositis (case 6). Comparable staining patterns and intensities of MHC‐II, MxA, and p62 were observed in both frozen (upper row) and FFPE (lower row) sections. Strong MHC‐II expression with sarcolemmal and sarcoplasmic staining was seen in the muscle fibres in both **(A)** frozen and **(D)** FFPE sections. MxA was negative in the muscle fibres in both **(B)** frozen and **(E)** FFPE sections. Strong p62 expression was present in the muscle fibres in both **(C)** frozen and **(F)** FFPE sections, exhibiting clumped dot‐like staining within rimmed vacuoles and diffuse fine granular staining in the sarcoplasm. All scale bars = 25 μm.

**Figure 2:**
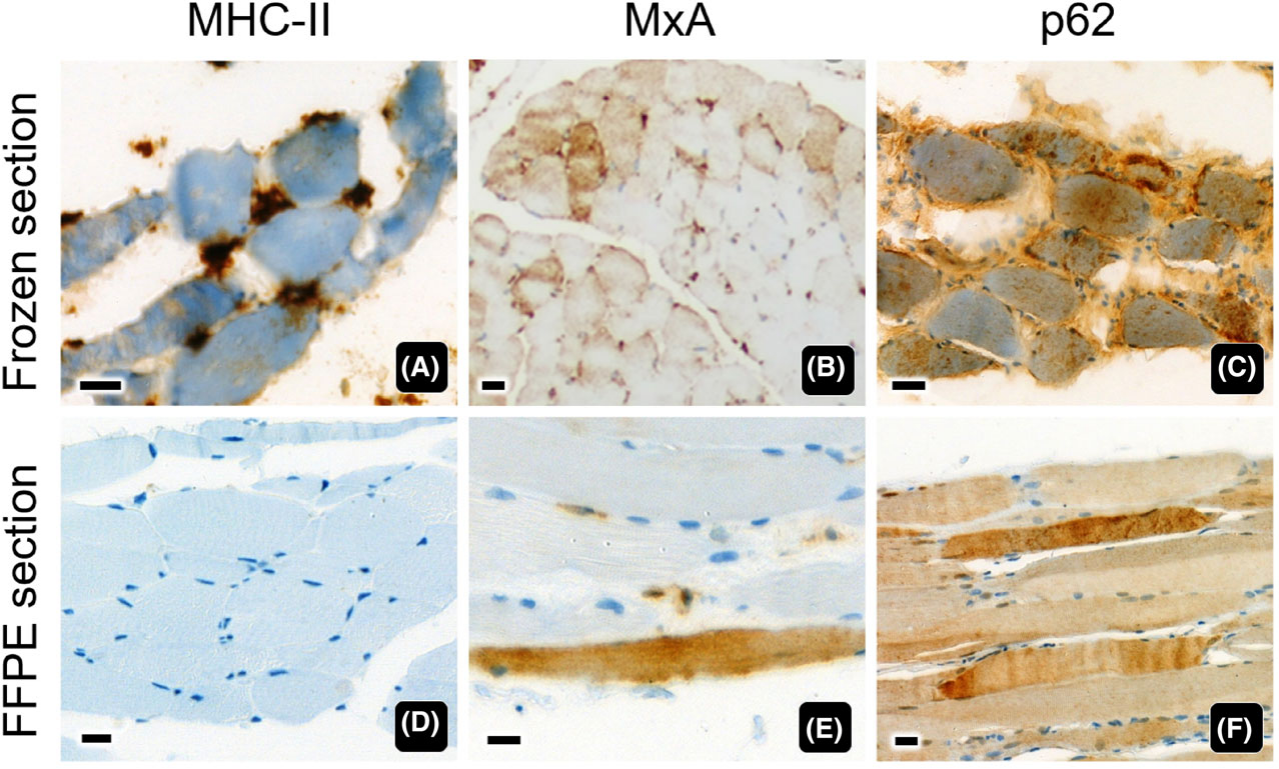
Dermatomyositis (case 1). Comparable staining patterns and intensities of MHC‐II, MxA, and p62 were observed in both frozen (upper row) and FFPE (lower row) sections. MHC‐II was negative in the muscle fibres in both **(A)** frozen and **(D)** FFPE sections. Moderate MxA expression was observed in the perifascicular atrophic muscle fibres in both **(B)** frozen and **(E)** FFPE sections. Moderate to strong p62 expression with diffuse fine granular sarcoplasmic staining was present in the muscle fibres in both **(C)** frozen and **(F)** FFPE sections. All scale bars = 25 μm.

**Figure 3:**
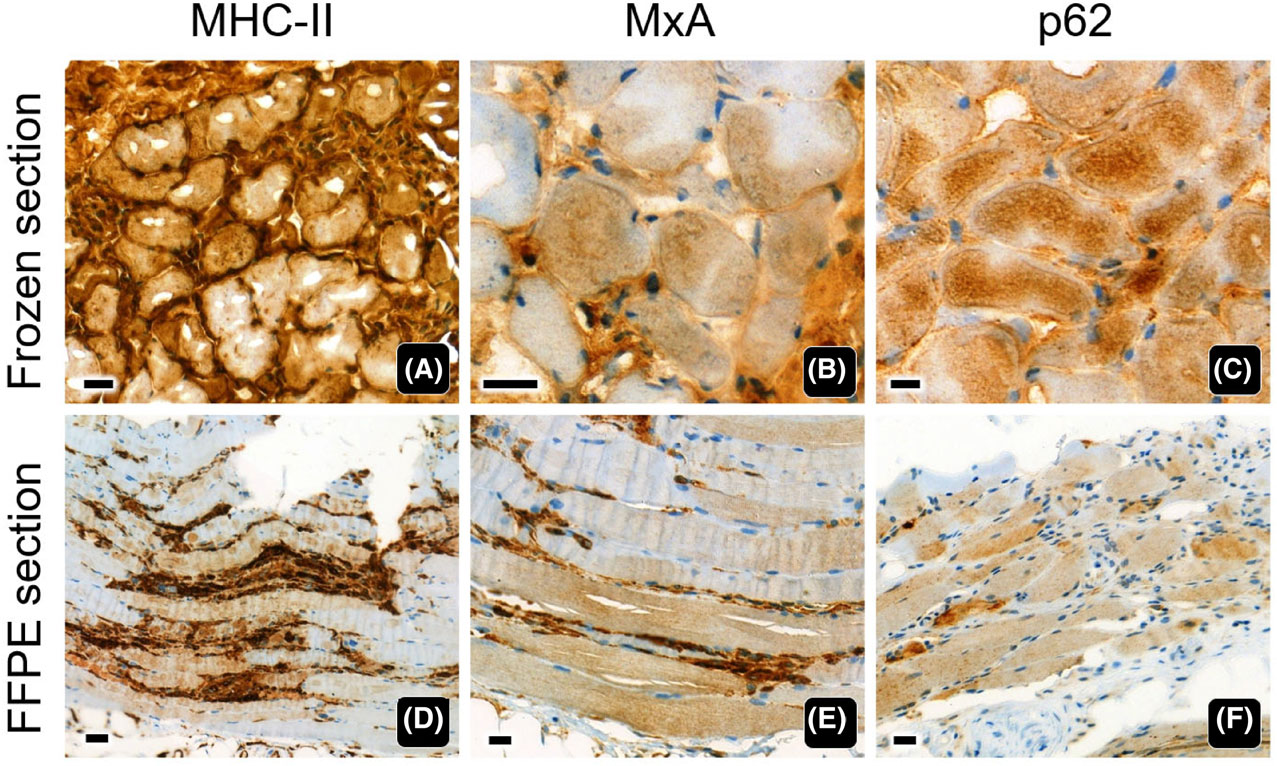
Immune‐mediated necrotizing myopathy (case 11). Comparable staining patterns and intensities of MHC‐II, MxA, and p62 were observed in both frozen (upper row) and FFPE (lower row) sections. Strong MHC‐II expression with sarcolemmal and sarcoplasmic staining was seen in the muscle fibres in both **(A)** frozen and **(D)** FFPE sections. Moderate MxA was observed in the muscle fibres in both **(B)** frozen and **(E)** FFPE sections. Strong p62 expression with diffuse fine granular sarcoplasmic staining was present in the muscle fibres in both **(C)** frozen and **(F)** FFPE sections. All scale bars = 25 μm.

Equivalent immunoreactivity staining for MHC‐II, MxA, and p62 was observed in 12 cases (Table 1). Representative photomicrographs from Case 6 (IBM), Case 1 (DM), and Case 11 (IMNM), illustrating these consistent findings, were presented in Figures 1, 2, 3, respectively. MHC‐II demonstrated clear sarcolemmal and sarcoplasmic immunoreactivity in FFPE sections (Figures 1D and 3D), matching the staining patterns observed in frozen sections (Figures 1A and 3A). MxA showed distinct sarcoplasmic staining in FFPE sections (Figures 2E and 3E) comparable to the frozen sections (Figures 1B and 3B). Similarly, p62 exhibited diffuse fine granular sarcoplasmic staining in FFPE sections (Figures 1F, 2F and 3F), consistent with the staining observed in frozen sections (Figures 1C, 2C and 3C). The immunohistochemical results in the FFPE sections were subsequently interpreted in the context of clinical information. In Case 6, the FFPE sections (Figure 1D–F) showed strong MHC‐II expression and clumped dot‐like staining within rimmed vacuoles for p62, along with absent MxA expression, which is consistent with the characteristic immunophenotype of IBM. In Case 1, the FFPE sections (Figure 2D–F) demonstrated perifascicular atrophy in longitudinal orientation, with positive MxA and p62 expression and absent MHC‐II staining, consistent with the typical immunophenotype of DM. In Case 11, the FFPE sections (Figure 3D–F) displayed MHC‐II, MxA, and p62 expression, consistent with the typical immunophenotype of IMNM.

**Figure 4:**
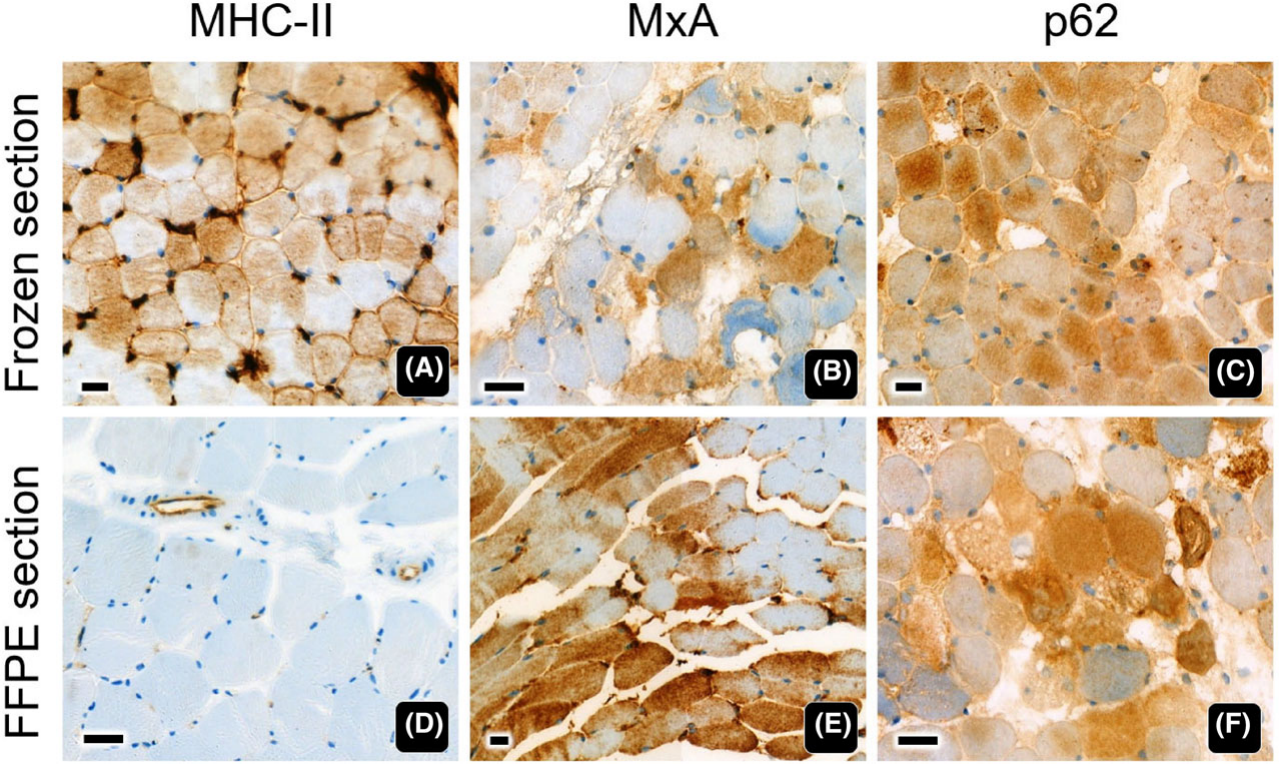
Juvenile dermatomyositis (case 5). **(A)** Moderate MHC‐II expression was seen in muscle fibres in the frozen section, **(D)** while no expression was detected in the FFPE section, with only internal positive staining in endothelial cells. Comparable staining patterns and intensities of MxA and p62 were observed in both frozen and FFPE sections. Strong MxA expression was present in the perifascicular atrophic muscle fibres in both **(B)** frozen and **(E)** FFPE sections. Strong p62 expression with diffuse fine granular sarcoplasmic staining was observed in muscle fibres in both **(C)** frozen and **(F)** FFPE sections. All scale bars = 25 μm.

**Figure 5:**
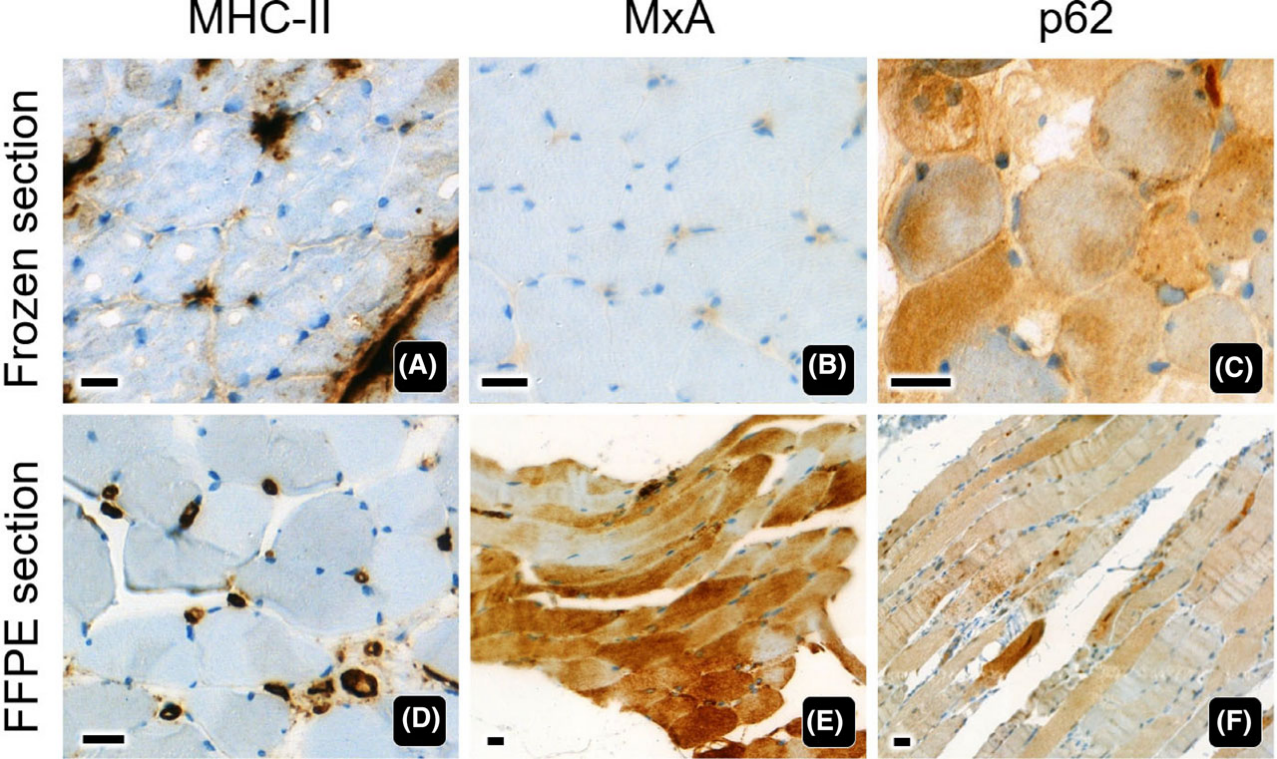
Dermatomyositis (case 3). MHC‐II expression was negative in muscle fibres, with only internal positive staining observed in endothelial cells in both **(A)** frozen and **(D)** FFPE sections. **(B)** MxA expression was absent in the frozen section, **(E)** but showed strong expression in the perifascicular atrophic muscle fibres in the FFPE section. Moderate p62 expression with diffuse fine granular sarcoplasmic staining was observed in muscle fibres in both **(C)** frozen and **(F)** FFPE sections. All scale bars = 25 μm.

Discrepancies in immunohistochemical staining between frozen and FFPE sections were observed in eight cases (Cases 2, 3, 5, 8, 9, 12, 15, 20), each involving a single marker (Table 1). Case 5 (juvenile DM) demonstrated MHC‐II positivity only in the frozen section (Figure 4A), with no staining in the FFPE section (Figure 4D). In contrast, Case 8 (IMNM) exhibited strong MHC‐II staining exclusively in the FFPE section but not in the frozen section. Case 3 (DM) showed strong MxA positivity exclusively in the FFPE section (Figure 5E) without staining in the frozen section (Figure 5B), whereas Case 12 (IMNM) exhibited moderate MxA staining in the frozen section with complete absence in the FFPE section. For p62, Case 2 (DM) and Case 15 (unspecified myopathy) displayed positivity only in the FFPE section, while Case 9 (IMNM) and Case 20 (neurogenic change of muscle) showed strong staining in the frozen section with negativity in the FFPE counterpart. To confirm these discrepant results, all corresponding archived frozen sections from the eight discordant cases were re‐examined and demonstrated identical staining findings to those observed in the frozen sections evaluated in the present study. These discordant IHC results may reflect the typical segmental and patchy antigen expression in IIM.

McNemar's test demonstrated no statistically significant differences in immunohistochemical positivity between frozen and FFPE muscle sections for all three markers (Table 3). For MHC‐II and MxA, only two discordant cases were observed for each marker, without significant difference between frozen and FFPE muscle sections (*P* = 0.4795). For p62, four discordant cases were identified. However, this difference also remained statistically non‐significant (*P* = 0.6171), indicating no systematic bias between frozen and FFPE sections. Overall concordance was high, reaching 90% for MHC‐II and MxA and 80% for p62. Collectively, these findings demonstrate that FFPE sections provide staining results comparable to frozen sections, supporting the reliability of FFPE tissue for assessing these markers in routine diagnostic practice.

**Table 3:** Concordance of MHC‐II, MxA, and p62 immunohistochemistry between frozen and FFPE muscle sections (*n* = 20), including *P*‐values

Markers	Concordant cases (%)			Discordant cases^a^ (%)			*P*‐value
	Frozen+/FFPE+	Frozen−/FFPE−	Total	Frozen+/FFPE−	Frozen−/FFPE+	Total	
MHC‐II	4 (20%)	14 (70%)	18 (90%)	1 (5%)	1 (5%)	2 (10%)	0.4795
MxA	3 (15%)	15 (75%)	18 (90%)	1 (5%)	1 (5%)	2 (10%)	0.4795
p62	16 (80%)	0 (0%)	16 (80%)	2 (10%)	2 (10%)	4 (20%)	0.6171

^a^ Discordant cases: MHC‐II (Cases 5, 8); MxA (Cases 3, 12); p62 (Cases 2, 9, 15, 20).

Comparison of MHC‐II, MxA, and p62 staining between frozen and FFPE muscle specimens yielded largely consistent positivity results across IIM subgroups, with minor variability in marker prevalence (Table 4). MHC‐II expression showed complete concordance in IBM (100% in both preparations) and absence in OM, while slightly lower detection rates were observed in DM (40% frozen vs. 20% FFPE) and a modest increase in IMNM using FFPE tissue (20% vs. 40%). MxA expression showed a similar overall distribution between frozen and FFPE sections. Positivity was higher in DM compared with other subgroups and was absent in IBM and OM in both preparations. Minor differences in prevalence were observed between frozen and FFPE tissues (DM: 40% vs. 60%; IMNM: 40% vs. 20%). p62 staining showed consistently high expression across all IIM subgroups and tissue types, particularly in IBM and OM (100% in both preparations), with only slight variation in DM and IMNM. Overall, these findings suggest that FFPE muscle sections provide comparable immunohistochemical detection of MHC‐II, MxA, and p62 relative to frozen specimens. This supports the feasibility of FFPE‐based evaluation in the diagnostic workup of IIM.

**Table 4:** Prevalence of MHC‐II, MxA, and p62 immunohistochemistry results between frozen and FFPE muscle sections among idiopathic inflammatory myopathy subgroups

Markers	Frozen muscle specimen				FFPE muscle specimen			
	DM (*n* = 5)	IBM (*n* = 2)	IMNM (*n* = 5)	OM (*n* = 1)	DM (*n* = 5)	IBM (*n* = 2)	IMNM (*n* = 5)	OM (*n* = 1)
MHC‐II	40% (2)	100% (2)	20% (1)	0% (0)	20% (1)	100% (2)	40% (2)	0% (0)
MxA	40% (2)	0% (0)	40% (2)	0% (0)	60% (3)	0% (0)	20% (1)	0% (0)
p62	80% (4)	100% (2)	100% (5)	100% (1)	100% (5)	100% (2)	80% (4)	100% (1)

## Discussion

In this study, we compared IHC staining outcomes for five diagnostic markers, MHC‐I, MHC‐II, MAC, MxA, and p62, between snap‐frozen and FFPE muscle biopsy specimens of 20 patients. The primary aim was to evaluate the feasibility of using FFPE sections in resource‐constrained settings lacking routine access to frozen IHC cuttings. Based on high concordance rates of 90% for both MHC‐II and MxA and 80% for p62, our findings indicate that FFPE muscle biopsies perform comparably to frozen sections in preserving antigenicity for these three immunohistochemical markers. FFPE sections of 3 μm were utilized in the current study because they enable superior morphological visualization of myofibers, whereas thicker 8‐μm FFPE sections in our previous study demonstrated overlapping sarcolemmal staining.^10^ Although data remain limited, available literature suggests that immunohistochemical protocols can be successfully optimized for FFPE muscle tissue when frozen sectioning is not feasible.^10^, ^12^, ^13^, ^14^ Suriyonplengsaeng *et al*. demonstrated successful FFPE‐based IHC utilization in muscular dystrophy, though limitations existed for certain epitopes.^10^ The findings in the present study build on this evidence in the context of IIM by demonstrating that MHC‐II, MxA, and p62 remain reliably detectable in FFPE samples. The observed discrepancy in 10–20% of cases (discordant staining), which did not reach statistical significance (*P* > 0.05), likely reflects the characteristic segmental and patchy involvement of IIM. The use of different cutting planes for the frozen and FFPE sections, although derived from the same muscle biopsy specimen, may also have contributed to the observed differences. These findings support that the discordance is attributable to biological heterogeneity and sampling variation rather than technical variability.

The second aim was to evaluate the diagnostic efficacy of using FFPE muscle specimens for IIM. In five DM cases, immunohistochemical assessment of MHC‐II, MxA, and p62 in FFPE sections demonstrated findings comparable to frozen tissue (Table 1). Notably, one DM case demonstrated strong perifascicular MxA positivity on FFPE sections despite absent staining in the corresponding frozen specimen (Figure 5). This observation underscores the robustness of FFPE‐based MxA IHC and highlights its ability to detect type I interferon‐associated changes critical for DM diagnosis. This finding is consistent with molecular evidence showing that *MX1* gene expression in muscle from active DM cases is profoundly elevated with an approximately 280‐fold increase relative to normal muscle reflecting the prominent type I interferon signature.^15^ MHC‐II expression provided supportive evidence in one to two cases of both preparations, consistent with previous reports demonstrating MHC‐II expression in 32%–55% of DM cases.^6^, ^16^ p62 staining was consistently positive but not disease‐specific. Milisenda *et al*. reported p62 expression in 57%, 87%, and 92% of DM, IMNM, and IBM cases, respectively, as well as in 26% of other myopathies.^17^ Immunohistochemical analysis of FFPE muscle specimens enabled definitive diagnosis of DM in three cases through perifascicular MxA expression and in one case through perifascicular MHC‐II expression (Table 1). The reported frequency of sarcoplasmic MxA expression in DM varies across studies, being identified in 46%, 71%, and 94% of cases in the cohorts of Waisayarat *et al*.,^18^ Uruha *et al*.,^11^ and Xing *et al*.,^19^ respectively. Soponkanaporn *et al*. reported sarcoplasmic MxA expression in 61% of juvenile DM cases.^20^ Despite this variability in prevalence, MxA is widely recognized as a relatively specific marker for DM, with specificity reaching 94% and 98% in the studies by Waisayarat *et al*.^18^ and Uruha *et al*.,^11^ respectively. Nevertheless, its sensitivity remains moderate, reported at 46% and 71% in these two studies. These findings indicate that absence of MxA expression does not exclude the diagnosis of DM. Indeed, MxA‐negative DM cases may be encountered, as exemplified by Case 17 and 5 in the present study.

In two IBM cases, diffuse MHC‐II expression and subsarcolemmal p62 aggregates were equally preserved in FFPE sections, mirroring frozen tissue findings and maintaining diagnostic interpretability (Table 1). These expression profiles in FFPE sections were sufficient to establish a definitive diagnosis in both cases when interpreted in conjunction with compatible clinical information. Diffuse MHC‐II expression is a well‐recognized feature of IBM, reported in 96% and 100% of cases by Lessard *et al*.^6^ and Rodríguez Cruz *et al*.,^16^ respectively. Although subsarcolemmal p62 aggregation is considered characteristic of IBM, this pattern has been reported in 28%, 55%, and 76% of DM, IMNM, and IBM cases, respectively.^17^ Milisenda *et al*. elucidated decreased p62 mRNA expression in IBM, suggesting that p62 protein accumulation is more likely attributable to impaired degradation than to enhanced synthesis.^17^ MxA expression was uniformly absent in both IBM cases across both preparations, consistent with the observations of Uruha *et al*., who demonstrated a complete lack of MxA staining in a cohort of 50 IBM cases.^11^

In five IMNM cases, immunohistochemical assessment of MHC‐II, MxA, and p62 in FFPE sections demonstrated patterns largely comparable to those observed in frozen tissue (Table 1). MHC‐II expression was present in only two IMNM cases evaluated using FFPE specimens, consistent with previous reports demonstrating MHC‐II expression in 17%–44% of IMNM cases.^6^, ^16^ MxA expression remained infrequent in IMNM across both tissue types, consistent with the absence of prominent type I interferon activation in this subtype and reinforcing its role in excluding DM rather than confirming IMNM. The frequency of sarcoplasmic MxA expression in IMNM was identified in only 2% and 8% of cases in the cohorts of Uruha *et al*.,^11^ and Waisayarat *et al*.,^18^ respectively. p62 staining was consistently detectable in FFPE specimens and paralleled frozen section findings, highlighting its reliability in demonstrating myofiber stress and degenerative changes.

In the OM case, MHC‐II and MxA expression were absent in both preparations, aligning with the expected immunopathologic profile of OM (Table 1). Although MHC‐II expression has been reported in up to 56%–85% of OM cases,^6^, ^16^ MxA expression appears to be uncommon, identified in only 14% of cases.^18^ p62 staining was consistently detectable across frozen and FFPE specimens, highlighting its robustness in demonstrating myofiber stress and degenerative changes rather than disease specificity.

MHC‐I IHC is considered non‐specific, being detected in 98% and 93% of IIM and non‐IIM groups, respectively.^16^ In contrast, the same study demonstrated MHC‐II expression in 62% of IIM cases compared with only 10% of non‐IIM cases, emphasizing its greater specificity relative to MHC‐I.^16^ Although MHC‐I staining is unsuccessful in FFPE specimens in this study, MHC‐II IHC can be reliably performed on FFPE tissue (Table 1). The ability to reliably perform MHC‐II IHC on FFPE muscle specimens, coupled with its higher specificity relative to MHC‐I, supports its use as a surrogate marker in routine diagnostic evaluation of IIM when frozen material is unavailable.

Capillary‐predominant MAC deposition is widely recognized as a typical histopathological feature of DM. In the study by Uruha *et al*., sarcoplasmic MxA demonstrated a higher sensitivity than capillary MAC deposition (71% vs. 35%), while maintaining comparable specificity (98% vs. 93%), indicating the potential value of MxA immunostaining in improving the pathological diagnosis of DM.^11^ Although MAC IHC failed to demonstrate staining in FFPE muscle specimens, MxA IHC was successfully applied to FFPE tissue (Table 1). Given its higher sensitivity, specificity, and technical feasibility in FFPE specimens, MxA serves as a practical and reliable adjunct marker for the routine evaluation of IIM when frozen material is unavailable.

The absence of MHC‐I and MAC immunoreactivity in this study is consistent with their validation restricted to frozen tissue sections and may reflect epitope masking or formalin‐induced antigen alteration in FFPE samples. The use of alternative FFPE‐validated antibodies may improve staining performance and further expand the applicability of FFPE muscle pathology. Nevertheless, further antibody optimization and evaluation were beyond the scope of the current study due to resource and time constraints. Despite the negative FFPE staining results for MHC‐I and MAC, our findings demonstrated that successful MHC‐II, MxA, and p62 immunostaining in FFPE muscle sections is sufficient to support the diagnosis of IIM in resource‐limited settings where frozen tissue processing is unavailable.

Several inflammatory markers relevant to muscle pathology, including CD3, CD20, CD4, CD8, CD56, and CD68, are well established for use in FFPE tissue. Our findings further demonstrate that MHC‐II, MxA, and p62 can be reliably detected in FFPE specimens. Taken together, these findings indicate that a substantial panel of diagnostically informative markers for IIM can be evaluated in FFPE muscle. FFPE tissue permits the preparation of thinner and more uniform sections (3 μm in FFPE sections compared with 10 μm in frozen sections), providing improved morphological resolution and clearer visualization of inflammatory cell distribution within muscle fibres, the endomysial, and perimysial compartments. This progression mirrors the historical shift in diagnostic pathology, particularly for lymphoma classification in hematopathology, where immunophenotypic characterization once dependent on frozen tissue is now routinely achieved using FFPE specimens following the advent of antigen retrieval and advances in immunohistochemical methodology.^21^, ^22^, ^23^

Collectively, these findings indicate that FFPE muscle tissue maintains comparable immunohistochemical performance to frozen sections for MHC‐II, MxA, and p62 in IIM diagnosis. Given the practical advantages of FFPE processing, including routine availability, standardized handling, and long‐term archival stability, FFPE‐based IHC represents a reliable and clinically useful alternative when frozen muscle specimens are limited or unavailable. The current absence of widely established FFPE IHC protocols for muscle biopsy highlights the urgent need to develop and standardize procedures specifically tailored to muscle pathology, particularly in resource‐constrained settings.

## Conclusions

FFPE muscle biopsy sections can reliably demonstrate immunohistochemical staining for MHC‐II, MxA, and p62 in most cases of IIM, with high concordance compared with frozen sections. Given the lack of statistically significant differences between modalities, FFPE‐based IHC offers a practical alternative in resource‐limited settings, facilitating accurate diagnosis without compromising diagnostic integrity. Moving forward, broader validation of FFPE protocols for IIM including standardized antigen retrieval methods and expanded marker panels is recommended to enhance diagnostic precision and accessibility. Additionally, larger studies are warranted to estimate more precisely the marker‐specific sensitivity and specificity of FFPE compared with frozen sections.

## Author contributions

JW conceived and designed the study. CS and JW reviewed and collected case data. JW examined histopathology. CS and JW evaluated IHC of FFPE and snapped frozen muscle tissue. CS drafted the manuscript. CS and JW provided a critique of the study, and revised the manuscript critically for important intellectual content. All authors have read and approved the final manuscript.

## Funding information

This research was funded by the Research Grant for Retrospective Study (Grant Type 5), Faculty of Medicine Ramathibodi Hospital, Mahidol University, Bangkok, Thailand (Grant No. R1106905001).

## Conflicts of interest

The authors declare no conflict of interest.

## Data Availability

The data and materials pertaining to this study are available from the corresponding author upon reasonable request.
